# Do physical activity and dietary smartphone applications incorporate evidence-based behaviour change techniques?

**DOI:** 10.1186/1471-2458-14-646

**Published:** 2014-06-25

**Authors:** Artur Direito, Leila Pfaeffli Dale, Emma Shields, Rosie Dobson, Robyn Whittaker, Ralph Maddison

**Affiliations:** 1 National Institute for Health Innovation, University of Auckland, 261 Morrin Rd, Auckland 1072, New Zealand; 2 National Institute for Health Innovation, School of Population Health, The University of Auckland, Private Bag 92019, Auckland Mail Centre, Auckland 1142, New Zealand

**Keywords:** Behaviour change techniques, Smartphone applications, Apps, Lifestyle, Physical activity, Diet, mHealth, Reliability

## Abstract

**Background:**

There has been a recent proliferation in the development of smartphone applications (apps) aimed at modifying various health behaviours. While interventions that incorporate behaviour change techniques (BCTs) have been associated with greater effectiveness, it is not clear to what extent smartphone apps incorporate such techniques. The purpose of this study was to investigate the presence of BCTs in physical activity and dietary apps and determine how reliably the taxonomy checklist can be used to identify BCTs in smartphone apps.

**Methods:**

The top-20 paid and top-20 free physical activity and/or dietary behaviour apps from the New Zealand Apple App Store Health & Fitness category were downloaded to an iPhone. Four independent raters user-tested and coded each app for the presence/absence of BCTs using the taxonomy of behaviour change techniques (26 BCTs in total). The number of BCTs included in the 40 apps was calculated. Krippendorff’s alpha was used to evaluate interrater reliability for each of the 26 BCTs.

**Results:**

Apps included an average of 8.1 (range 2-18) techniques, the number being slightly higher for paid (M = 9.7, range 2-18) than free apps (M = 6.6, range 3-14). The most frequently included BCTs were “provide instruction” (83% of the apps), “set graded tasks” (70%), and “prompt self-monitoring” (60%). Techniques such as “teach to use prompts/cues”, “agree on behavioural contract”, “relapse prevention” and “time management” were not present in the apps reviewed. Interrater reliability coefficients ranged from 0.1 to 0.9 (Mean 0.6, SD = 0.2).

**Conclusions:**

Presence of BCTs varied by app type and price; however, BCTs associated with increased intervention effectiveness were in general more common in paid apps. The taxonomy checklist can be used by independent raters to reliably identify BCTs in physical activity and dietary behaviour smartphone apps.

## Background

Lifestyle behaviours, such as diet and physical activity, are modifiable risk factors associated with many non-communicable diseases (NCDs), which account for 63% of deaths worldwide [1]. To date, many intervention programs targeting physical activity and dietary changes have had modest effects and their long-term effectiveness is not well established [2-5]. Thus, public health researchers have begun to examine novel approaches to deliver behaviour change interventions. Mobile and wireless technology (mHealth) is a growing area in the prevention and management of NCDs and holds potential to deliver health-related behaviour change interventions [6-8]. Mobile phone ownership has reached saturation in many developed countries with an increase in smartphone ownership. A 2012 survey in the United States (U.S.) of three thousand adults indicated that 85% owned a mobile phone, 53% of those being smartphones. Moreover, 84% of smartphone owners had downloaded an app to their device and 19% had downloaded an app to specifically manage their health [9].

Despite the recent proliferation of apps to promote positive lifestyle change, there is a dearth of research evidence regarding their effectiveness. Further, content analysis of existing apps have identified gaps between evidence based guidelines and app content relating to smoking cessation [10,11], weight loss [12,13], diabetes [14] and exercise [15]. While theoretically grounded mHealth behaviour interventions increase the likelihood of achieving behaviour change, it has been suggested that current theories are inadequate to guide mHealth interventions, which need to be more interactive and dynamic [16]. Abraham and Michie have suggested that there are a number of behaviour change techniques (BCTs) common to many health behaviour theories [17], of which at least five are evident in effective physical activity and dietary interventions (i.e. self-monitoring, intention formation, specific goal setting, review of behavioural goals and feedback on performance) [18]. While studies have been conducted to determine the extent to which behaviour change theory has been applied to app development, none have quantified the extent to which specific BCTs are included.

The present study sought to determine the presence/absence of BCTs in the top 20 free and top 20 paid physical activity and dietary smartphone apps from the New Zealand iTunes Apple App Store Health & Fitness category. A second aim was to determine whether the BCTs taxonomy checklist could be used to reliably identify BCTs in smartphone apps.

## Methods

### Study design

A comparative assessment of the presence of BCTs within smartphone apps from the New Zealand Apple App Store Health & Fitness category was undertaken by four independent raters. The raters were all trained in health behaviour change; one dietetics and nutrition masters student, two health sciences doctoral students and one health psychologist. The study was conducted in accordance to ethical standards. Human subjects were not recruited and therefore no ethics approval was required.

### Sample

**Figure 1:**
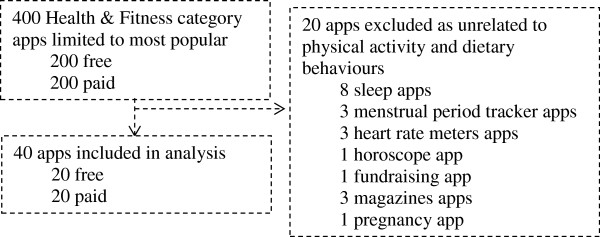
**Selection of sample of apps.** Procedure for selection of sample of physical activity and dietary apps.

The apps were located and downloaded using the software ‘iTunes’ on November 12 of 2012 (available for download at http://www.apple.com/itunes). To be included in the present evaluation apps had to be designed either to promote health or prevent disease, and specifically address physical activity and/or dietary behaviours. Irrelevant and miscategorised apps under the specified Health & Fitness category or apps addressing other health behaviours were excluded. Apps were identified within the Health & Fitness category of iTunes by clicking on the “Top” ordering function button (i.e. “Top Paid iPhone Apps” and “Top Free iPhone Apps”). Their titles and descriptions were initially screened by the first author. Apps that met the inclusion criteria were downloaded until a total of 20 was achieved. This procedure was repeated to retrieve the 20-top paid and 20-top free apps. During screening, nine top-paid and 11 top-free apps were excluded for not meeting the inclusion criteria (see Figure 1 for an overview of the sampling procedure). All apps were downloaded to an iPhone 4 or 5 running iOS 6.0.1 (version of Apple operating system for iPhones).

### Measurement

The Taxonomy of Behaviour Change Techniques Used in Interventions and the Coding Manual to Identify Behaviour Change Techniques in Behaviour Change Intervention Descriptions were used for the present evaluation [17]. Abraham and Michie previously developed and demonstrated the feasibility and reliability of using the taxonomy for identifying BCTs in behavioural interventions. The coding manual provides guidelines to detect whether an intervention description includes any or all of the 26 defined BCTs. Mean kappa values of 0.80 and 0.82 (i.e. good reliability) have been observed when applying the taxonomy of BCTs to physical activity and healthy eating intervention descriptions, respectively [17]. Each app was rated for inclusion of each of the 26 BCTs. Formal statistical comparisons in terms of differences in the number of BCTs between apps were not conducted for the following reasons. First this was an exploratory study to determine whether an existing coding system could be used reliably to assess BCTs among mobile phone apps. Second, given the number of apps assessed in this study and the potential for differences in BCTs between apps, the number of comparisons needed would likely result in Type 1 error.

### Procedure

For each app, descriptive information was retrieved regarding its popularity (i.e. frequency of downloads relative to other apps within the same category), average rating (i.e. average number of stars the app was rated ranging one to five), total ratings (i.e. number of users who downloaded the app and voluntarily rated it), total "hate it", “don’t like it”, “it’s ok”, “it’s good”, and “it’s great” ratings (i.e. number of times the app was rated with one to five stars, respectively), customer reviews (i.e. number of times the app was reviewed) and price (as shown in Additional file 1). Every app was evaluated by four independent raters using three iPhones between November 2012 and April 2013. The four raters separately tested all apps in detail to become familiar with the interfaces, menus, features, and functionality (e.g. “profile”, “routes”, “workouts”, “friends”, “meals”, “charts”, “analysis”, “my plan”, “nutrition”, “settings”, “tips & tricks”, “FAQ”). The apps were user-tested independently by each rater.

Prior to evaluation, all raters read the BCTs definitions carefully and had the opportunity to clarify and discuss the definitions. Before beginning a coding session raters read each BCT description carefully to ensure clear differentiation between techniques. After using each app, raters reviewed each of the menu functions to rate the presence or absence of BCTs according to the checklist. A dichotomous score of “0” absent or “1” present was applied for each of the 26 BCTs. Disagreements were resolved by consensus discussion.

### Statistical analyses

All statistical analyses were conducted using IBM SPSS Statistics version 20.0. Frequencies and percentages of each of the 26 BCTs included in the 40 apps were calculated. Krippendorff’s alpha was used to evaluate interrater reliability for each of the 26 BCTs. This statistic is appropriate because it can be used with any number of observers, sample sizes, and satisfies all criteria for a good measure of reliability [19]. Further, a macro that computes Krippendorff’s alpha is available for statistical software packages, such as SPSS [19].

## Results

**Figure 2:**
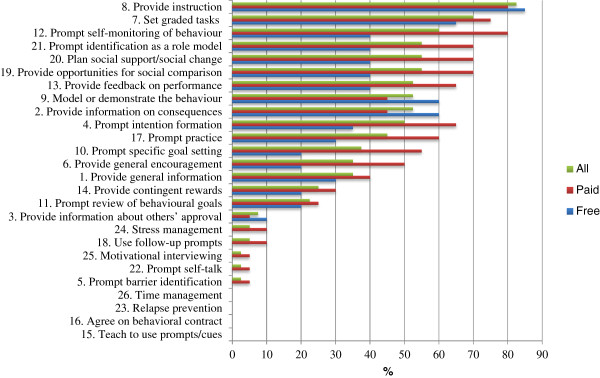
**Incorporation of BCTs within the top-40 apps.** Percentage of apps incorporating each of the 26 BCTs within the Top-40 Applications from the New Zealand Apple App Store Health & Fitness category by cost and overall.

The majority of the 40 apps reviewed targeted physical activity (30 apps, 75%), followed by dietary behaviour (6 apps, 15%), and combined behaviours (4 apps, 10%). Attributes of the paid and free apps are presented in Table 1. Generally, apps were rated in iTunes customer ratings as good (mean = 4.1 on a scale of one to five stars). Free apps were on average rated more times (193.4) and had more customer reviews (108.7) than paid ones (35.9 and 22.9, respectively). The average rating was slightly higher for paid (4.2) compared to free apps (3.9).Overall, apps included an average of 8.1 (range 2-18) BCTs, with slightly more BCTs present for paid (mean = 9.7, range 2-18) as compared to free apps (mean = 6.6, range 3-14) (see Figure 2). Commonly included BCTs were “provide instruction” (83% of the apps), “set graded tasks” (70%), and “prompt self-monitoring” (60%). “Model/demonstrate the behaviour” (53%), “provide opportunities for social comparison”, “plan social support/social change” and “prompt identification as a role model” were also frequently incorporated (55%). “Prompt barrier identification”, “prompt self-talk”, and “motivational interviewing” were seldom included (3%), and “teach to use prompts/cues”, “agree on behavioural contract”, “relapse prevention” and “time management” were not included. “Prompt intention formation”, “provide general encouragement”, “prompt specific goal setting”, “prompt self-monitoring of behaviour”, and “prompt practice” were techniques included more frequently in paid compared to free apps.

**Table 1:** Descriptive data of the top-40 apps

**Attributes**	**Free**	**Paid**	**Overall**
Average rating (1-5)	3.93	4.17	4.05
	(0.55)	(0.94)	(0.77)
	[3-5]	[1-5]	[1-5]
Total ratings	193.35	35.85	114.60
	(567.78)	(35.54)	(405.00)
	[0-2529]	[0-115]	[0-2529]
"hate it" ratings (1 star)	13.00	1.80	7.40
	(24.75)	(2.38)	(18.25)
	[0-92]	[0-9]	[0-92]
“don’t like it” ratings (2 stars)	5.80	1.20	3.50
	(11.72)	(1.40)	(8.56)
	[0-47]	[0-4]	[0-47]
“it’s ok” ratings (3 stars)	8.80	2.35	5.58
	(20.42)	(3.41)	(14.81)
	[0-79]	[0-14]	[0-79]
“it’s good” ratings (4 stars)	35.60	8.25	21.93
	(90.82)	(10.09)	(65.27)
	[0-371]	[0-32]	[0-371]
“it’s great” ratings (5 stars)	130.15	22.25	76.20
	(430.92)	(23.05)	(306.12)
	[0-1940]	[0-74]	[0-1940]
Customer reviews	108.70	22.90	65.80
	(341.48)	(27.14)	(243.01)
	[0-1527]	[0-89]	[0-1527]
Price (NZD$)	-	3.28	1.64
		(2.35)	(2.33)
		[1.29-10.99]	[0-10.99]
Total BCTs	6.55	9.65	8.10
	(3.50)	(4.38)	(4.22)
	[3-14]	[2-18]	[2-18]
PA behaviour apps	15	15	30
Dietary behaviour apps	3	3	6
PA + Dietary behaviour apps	2	2	4

Descriptive data of the top-40 apps from the New Zealand Apple App Store Health & Fitness category (Mean, (Standard Deviation), [Range]).

*Note.* Standard Deviations appear in parentheses below means; Ranges appear in brackets below SD. PA = Physical Activity; BCTs = Behaviour Change Techniques.

Reliability data are presented in Table 2. Coefficients ranged from 0.1 to 0.9 with a mean of 0.6 (SD = 0.2), indicating moderate reliability. Raters agreed that none of the apps used BCT15 “teach to use prompts/cues”, BCT16 “agree on behavioural contract”, BCT23 “relapse prevention” and BCT26 “time management”. Despite 100% agreement, the calculation of alphas for these techniques was not possible because there was no variation in the reliability data matrix [19]. Of the 22 reliability tests, seven (32%) yielded alphas of more than 0.7, indicating good reliability, and only two (9%) were below 0.4. Inferior reliability was observed for four techniques: BCT2 “provide information on consequences” (0.4), BCT3 “provide information about others’ approval” (0.4), BCT5 “prompt barrier identification” (0.1), which was only observed once, and BCT6 “provide general encouragement” (0.4).

**Table 2:** Reliability of BCT identification

**Technique**	**Krippendorf’s α**
1. Provide general information	.57
2. Provide information on consequences	.41
3. Provide information about others’ approval	.38
4. Prompt intention formation	.57
5. Prompt barrier identification	.10
6. Provide general encouragement	.41
7. Set graded tasks	.81
8. Provide instruction	.66
9. Model or demonstrate the behaviour	.82
10. Prompt specific goal setting	.52
11. Prompt review of behavioural goals	.47
12. Prompt self-monitoring of behaviour	.85
13. Provide feedback on performance	.54
14. Provide contingent rewards	.60
15. Teach to use prompts/cues	*
16. Agree on behavioural contract	*
17. Prompt practice	.56
18. Use follow-up prompts	.46
19. Provide opportunities for social comparison	.88
20. Plan social support/social change	.88
21. Prompt identification as a role model	.90
22. Prompt self-talk	.66
23. Relapse prevention	*
24. Stress management	.85
25. Motivational interviewing	.66
26. Time management	*
Mean	.62

Reliability of BCT identification in the top-40 apps from the New Zealand Apple App Store Health & Fitness category: Krippendorf’s alpha per technique.

*Note*. *- Input reliability data matrix exhibits no variation. BCTs = Behaviour Change Techniques.

## Discussion

This study identified the presence or absence of BCTs in popular physical activity and/or dietary behaviour apps. There was substantial variation in the numbers of BCTs present, with an average of eight techniques per app. Using a taxonomy and coding manual it was possible to identify BCTs used in smartphone health behaviour change applications. Beyond these general observations, specific issues are outlined below.

Previous research has already highlighted the shortage of theoretical content present in interactive technologies such as web sites [20] and apps [10,12,14] designed to promote health behaviour change [11,13,15,21,22]. Consistent with previous research, our findings demonstrate the relative absence of behaviour change strategies present in physical activity and dietary apps. Moreover, this study highlights the potential to improve future app development by incorporating key strategies known to enhance behaviour change. For example, existing technologies permit real time assessment, feedback, and tailoring, however, in the present study, only 38% and 23% of the apps prompted specific goal setting or prompted review of behavioural goals, respectively.

The five BCTs shown to be commonly associated with greater effectiveness for modifying physical activity and diet in previous studies were present to varying degrees in the apps reviewed here (i.e. self-monitoring – 60% of apps, intention formation – 50%, specific goal setting – 38%, review of behavioural goals – 23% and feedback on performance – 53%). However, these five BCTs were in general more common in paid versus free apps. BCTs such as relapse prevention, which is important for sustained behavioural change [23] was not present in any of the reviewed apps, which questions the value of these apps for changing behaviour in the long-term.

The observed differences in reliability identifying BCTs indicate the need to clarify definitions and/or coding instructions. We evaluated the presence of BCTs using a coding instrument originally developed to identify BCTs from written text in published papers describing an intervention [17]. Perhaps specific coding instructions to apply when assessing the active ingredients of mHealth or interactive technologies such as apps or video games can be developed. The present research included a taxonomy of 26 techniques; however, subsequent taxonomies have been developed [24]. Future content analysis of apps should apply this updated hierarchical version of the BCT taxonomy.

While identifying the active content of health behaviour change interventions is crucial, researchers must be aware of the caveats of ascribing effectiveness to certain BCTs or combinations of BCTs. To do so, researchers should also consider the parameters for effectiveness for each BCT. These are the required characteristics that a translation of a BCT to usable intervention elements must incorporate (i.e., an effective BCT is undermined if not correctly applied) [25]. Additionally, the effectiveness of BCTs is determined by contextual factors such as target population (e.g., sample characteristics), behavioural domain (e.g., physical activity, smoking) and study design factors (e.g., follow-up period, blinding). Further, BCTs frequently co-occur in interventions and they can interact with each other [25]. Hence, conclusions about the behaviour change potential of apps based on incorporation of BCTs should be interpreted cautiously as BCTs are not effective under all conditions. Caution interpreting our findings in terms of differences in the number of BCTs between apps is also warranted as we did not conduct formal statistical comparisons.

The increasing number and diversity of apps available makes its assessment a difficult task for the public and clinicians to differentiate which apps can be useful in promoting behaviour change. Presumably, the value of apps can be enhanced by developers incorporating more features, theory, and BCTs into their apps, which in turn will increase the behaviour change potential of the app. The current study suggests the higher potential quality of paid apps should be a factor to weigh when selecting and using apps for personal use, clinical intervention, or future research. Furthermore, guidelines can be created to influence and help app developers as to which BCTs (and other components) to include that likely will enhance the behaviour change potential of apps.

Despite the proliferation of physical activity and dietary apps, it is not clear whether they are effective at modifying behaviour. At present, there is a dearth of effectiveness data of app-based interventions to promote healthy behaviours [26], and robust, rigorously conducted and adequately powered trials are required to determine their effectiveness. On the other hand, app development proceeds at a rate that far out paces time frames typically observed in trial development and conduct. Thus, more dynamic forms of evaluation methods are required to determine the effectiveness of such technologies [27]. Generally, the effectiveness of mHealth interventions such as text messaging for modifying health behaviours (e.g., smoking cessation) has been established [28]; however the effectiveness of more complex and dynamic mHealth interventions including apps has yet to be determined.

A strength of this study was the use of an established instrument to systematically rate the incorporation of BCTs in the respective apps. However, in the present study, the presence of BCTs was determined by user-testing the apps rather than from text descriptions. Some app features were not explicit during use. For example, reminders, weekly updates, and pop-up feedback, etc, may have occurred for one, but not all raters at any given time. Despite these issues, modest reliability between raters was observed (0.6). Another strength was the use of four raters, with a range of behaviour change experiences, which provided a more comprehensive assessment of the apps and the use of the taxonomy checklist. A major limitation of this study was not including apps from other app stores such as the Google Play Store/Android platform, or app stores from other countries besides NZ, which limits the generalisability of the findings. Nevertheless, we investigated the most popular and commonly downloaded apps of the iTunes Apple App Store Health & Fitness category, which represent a sample of apps that many people are using and therefore increases the study relevance. Of note, apps may exist that incorporate more evidence based BCTs than those included in the study sample as we only rated the most popular apps. Furthermore, technology has a dynamic nature with new apps and updates developed every day, consequently, these evaluations need to be updated periodically.

The advantages of mobile phone (mHealth) solutions compared to other health intervention delivery modes include the persistent interactivity, personalisation and engagement, potential to make healthcare more accessible and scalable, more cost-effective and more equitable [29]. Such characteristics provide significant potential to assist in disease prevention strategies and supporting sustained change in lifestyle behaviours. However, there are too many apps for consumers and professionals to choose from [30]. In addition, the majority within the health & fitness category of the Apple iTunes U.S. store scored less than 40 out of a possible 100 for functionality according to a recent report from the IMS Institute for Healthcare Informatics that concluded apps do little more than providing information [31]. Emerging evidence demonstrates the need for collaboration between health behaviour change experts and app developers to create apps that include effective BCTs. Future research is also needed to better understand how individuals use apps after downloading them, and to investigate features that may impact user acceptability and preference [32].

## Conclusions

Presence of BCTs varied by app type and price; however, BCTs associated with increased intervention effectiveness were in general more common in paid apps. The BCTs taxonomy checklist can be used by independent raters to reliably identify BCTs in physical activity and dietary behaviour smartphone apps.

## Competing interests

Author Direito, Author Dale, Author Shields, Author Dobson, Author Whittaker and Author Maddison declare that they have no conflict of interest. All authors have no financial disclosures.

## Authors’ contributions

AD contributed to the conception and design of the study, performed the statistical analysis, interpreted the data, and led the writing of the paper. AD, LPD, ES and RD contributed to acquisition of data. LPD, ES, RD, RW and RM participated in the conceptualisation of the study, helped to interpret the data and provided substantive feedback on the manuscript. All authors have read and approved the final manuscript.

## Pre-publication history

The pre-publication history for this paper can be accessed here:

http://www.biomedcentral.com/1471-2458/14/646/prepub

## Supplementary Material

Additional file 1 **Characteristics Of The Apps.** Characteristics Of The Study Sample Of Physical Activity And Dietary Apps. Microsoft Word Document. This table provides the characteristics of the study sample of apps. Click here for file

## References

[B1] World Health Organization (WHO) Global Status Report On Noncommunicable Diseases 2010 2010

[B2] Brown T Avenell A Edmunds LD Moore H Whittaker V Avery L Summerbell C For the PT: Systematic review of long-term lifestyle interventions to prevent weight gain and morbidity in adults Obes Rev 2009 10 6 627 638 19754634 10.1111/j.1467-789X.2009.00641.x

[B3] Foster C Hillsdon M Thorogood M Kaur A Wedatilake T Interventions for promoting physical activity Cochrane Database Syst Rev 2005 1 CD003180 15674903 10.1002/14651858.CD003180.pub2 PMC4164373

[B4] Oude Luttikhuis H Baur L Jansen H Shrewsbury VA O'Malley C Stolk RP Summerbell CD Interventions for treating obesity in children Cochrane Database Syst Rev 2009 1 CD001872 19160202 10.1002/14651858.CD001872.pub2

[B5] Waters E de Silva-Sanigorski A Burford BJ Brown T Campbell KJ Gao Y Armstrong R Prosser L Summerbell Carolyn D Interventions for preventing obesity in children Cochrane Database Syst Rev 2011 12 CD001871 22161367 10.1002/14651858.CD001871.pub3

[B6] Free C Phillips G Galli L Watson L Felix L Edwards P Patel V Haines A The effectiveness of mobile-health technology-based health behaviour change or disease management interventions for health care consumers: a systematic review PLoS Med 2013 10 1 1 45 10.1371/journal.pmed.1001362 PMC3548655 23349621

[B7] Kumar S Nilsen WJ Abernethy A Atienza A Patrick K Pavel M Riley WT Shar A Spring B Spruijt-Metz D Hedeker D Honavar V Kravitz R Lefebvre RC Mohr DC Murphy SA Quinn C Shusterman V Swendeman D Mobile health technology evaluation: the mhealth evidence workshop Am J Prev Med 2013 45 2 228 236 23867031 10.1016/j.amepre.2013.03.017 PMC3803146

[B8] Steinhubl SR Muse ED Topol EJ Can mobile health technologies transform health care? JAMA 2013 310 22 2395 2396 24158428 10.1001/jama.2013.281078

[B9] Mobile Health 2012 http://pewinternet.org/Reports/2012/Mobile-Health.aspx

[B10] Abroms LC Padmanabhan N Thaweethai L Phillips T iPhone apps for smoking cessation: a content analysis Am J Prev Med 2011 40 3 279 285 21335258 10.1016/j.amepre.2010.10.032 PMC3395318

[B11] Abroms LC Lee Westmaas J Bontemps-Jones J Ramani R Mellerson J A content analysis of popular smartphone apps for smoking cessation Am J Prev Med 2013 45 6 732 736 24237915 10.1016/j.amepre.2013.07.008 PMC3836190

[B12] Breton ER Fuemmeler BF Abroms LC Weight loss—there is an app for that! But does it adhere to evidence-informed practices? Transl Behav Med 2011 1 4 523 529 24073074 10.1007/s13142-011-0076-5 PMC3717669

[B13] Pagoto S Schneider K Jojic M DeBiasse M Mann D Evidence-based strategies in weight-loss mobile apps Am J Prev Med 2013 45 5 576 582 24139770 10.1016/j.amepre.2013.04.025

[B14] Chomutare T Fernandez-Luque L Årsand E Hartvigsen G Features of mobile diabetes applications: review of the literature and analysis of current applications compared against evidence-based guidelines J Med Internet Res 2011 13 3 e65 21979293 10.2196/jmir.1874 PMC3222161

[B15] Cowan LT Van Wagenen SA Brown BA Hedin RJ Seino-Stephan Y Hall PC West JH Apps of steel: are exercise apps providing consumers with realistic expectations? A content analysis of exercise apps for presence of behavior change theory Health Educ Behav 2013 40 2 133 139 22991048 10.1177/1090198112452126

[B16] Riley W Rivera D Atienza A Nilsen W Allison S Mermelstein R Health behavior models in the age of mobile interventions: are our theories up to the task? Transl Behav Med 2011 1 1 53 71 21796270 10.1007/s13142-011-0021-7 PMC3142960

[B17] Abraham C Michie S A taxonomy of behavior change techniques used in interventions Health Psychol 2008 27 3 379 387 18624603 10.1037/0278-6133.27.3.379

[B18] Michie S Abraham C Whittington C McAteer J Gupta S Effective techniques in healthy eating and physical activity interventions: a meta-regression Health Psychol 2009 28 6 690 701 19916637 10.1037/a0016136

[B19] Hayes AF Krippendorff K Answering the call for a standard reliability measure for coding data Commun Meth Meas 2007 1 1 77 89

[B20] Doshi A Patrick K Sallis JF Calfas K Evaluation of physical activity web sites for use of behavior change theories Ann Behav Med 2003 25 2 105 111 12704012 10.1207/S15324796ABM2502_06

[B21] West JH Hall PC Hanson CL Barnes MD Giraud-Carrier C Barrett J There’s an app for that: content analysis of paid health and fitness apps J Med Internet Res 2012 14 3 e72 22584372 10.2196/jmir.1977 PMC3799565

[B22] Azar KMJ Lesser LI Laing BY Stephens J Aurora MS Burke LE Palaniappan LP Mobile applications for weight management: theory-based content analysis Am J Prev Med 2013 45 5 583 589 24139771 10.1016/j.amepre.2013.07.005

[B23] Williams SL French DP What are the most effective intervention techniques for changing physical activity self-efficacy and physical activity behaviour—and are they the same? Health Educ Res 2011 26 2 308 322 21321008 10.1093/her/cyr005

[B24] Michie S Richardson M Johnston M Abraham C Francis J Hardeman W Eccles MP Cane J Wood CE The behavior change technique taxonomy (v1) of 93 hierarchically clustered techniques: building an international consensus for the reporting of behavior change interventions Ann Behav Med 2013 46 1 81 95 23512568 10.1007/s12160-013-9486-6

[B25] Peters G-JY de Bruin M Crutzen R Everything should be as simple as possible, but no simpler: towards a protocol for accumulating evidence regarding the active content of health behaviour change interventions Health Psychol Rev 2013 Advance online publication. doi:10.1080/17437199.2013.848409 10.1080/17437199.2013.848409 PMC4376231 25793484

[B26] Glynn L Hayes P Casey M Glynn F Alvarez-Iglesias A Newell J ÓLaighin G Heaney D Murphy A SMART MOVE - a smartphone-based intervention to promote physical activity in primary care: study protocol for a randomized controlled trial Trials 2013 14 1 1 7 23714362 10.1186/1745-6215-14-157 PMC3680242

[B27] Mohr DC Cheung K Schueller SM Hendricks Brown C Duan N Continuous evaluation of evolving behavioral intervention technologies Am J Prev Med 2013 45 4 517 523 24050429 10.1016/j.amepre.2013.06.006 PMC3828034

[B28] Whittaker R McRobbie H Bullen C Borland R Rodgers A Gu Y Mobile phone-based interventions for smoking cessation Cochrane Database Syst Rev 2012 11 CD006611 23152238 10.1002/14651858.CD006611.pub3

[B29] Whittaker R Key issues in mobile health and implications for New Zealand Health Care Inform Rev Onlin 2012 16 2 2 7

[B30] van Velsen L Beaujean DJ van Gemert-Pijnen JE Why mobile health app overload drives us crazy, and how to restore the sanity BMC Med Inform Decis Mak 2013 13 1 23 23399513 10.1186/1472-6947-13-23 PMC3621678

[B31] Aitken M Gauntlett C IMS Institute for Healthcare Informatics Patient Apps for Improved Healthcare: From Novelty to Mainstream 2013 NJ, USA: IMS Institute for Healthcare Informatics

[B32] Dennison L Morrison L Conway G Yardley L Opportunities and challenges for smartphone applications in supporting health behavior change: qualitative study J Med Internet Res 2013 15 4 e86 23598614 10.2196/jmir.2583 PMC3636318

