# Supplementary material for: Do physical activity and dietary smartphone applications incorporate evidence-based behaviour change techniques?
Source: BMC Public Health. 2014 Jun 25;14:646. doi: 10.1186/1471-2458-14-646 (PMC4080693; doi:10.1186/1471-2458-14-646)
Supplement: Additional file 1 — Characteristics Of The Apps. Characteristics Of The Study Sample Of Physical Activity And Dietary Apps. Microsoft Word Document. This table provides the characteristics of the study sample of apps. [file 1471-2458-14-646-S1.docx]

# **Additional Files**

## **Additional File 1 - Characteristics Of The Apps**

Additional File 1 - Characteristics Of The Study Sample Of Physical Activity And Dietary Apps

Microsoft Word Document

This table provides the characteristics of the study sample of apps.

| *Characteristics Of The Study Sample Of Physical Activity And Dietary Apps* | | | | | |
| --- | --- | --- | --- | --- | --- |
| App name | Developer | BCTs total | Popularity | Average ratings | Price (NZD$) |
| Jillian Michaels Slim-Down Solution - Diet, Fitness, Exercise Advice | Everyday Health, Inc. | 18 | 12 | 4 | 1.29 |
| All-in Fitness: 1000 Exercises, Workouts & Calorie Counter | Arawella Corporation | 15 | 8 | 4.5 | 1.29 |
| Calorie Counter* | About, Inc. | 14 | 13 | 4 | 0 |
| iDukan Diet Tracker | Harptree Software Ltd | 14 | 10 |  | 5.29 |
| Calorie Counter PRO by MyNetDiary | MyNetDiary Inc. | 13 | 5 | 4.5 | 5.29 |
| Calorie Counter by MyNetDiary | MyNetDiary Inc. | 12 | 7 | 3.5 | 0 |
| RunKeeper - GPS Track Running Walking Cycling | FitnessKeeper, Inc | 12 | 12 | 4.5 | 0 |
| Hundred PushUps | SoftwareX | 12 | 23 | 4 | 2.59 |
| 5K Runner: 0 to 5K run training | Clear Sky Apps | 12 | 9 | 5 | 4.19 |
| Zombies, Run! 5k Training | Six to Start and Naomi Alderman | 12 | 11 |  | 5.29 |
| Zombies, Run! | Six to Start | 12 | 16 | 4.5 | 10.99 |
| Nike Training Club | Nike, Inc | 11 | 14 | 5 | 0 |
| All-in Yoga: 300 Poses & Yoga Classes | Arawella Corporation | 11 | 4 | 4 | 1.29 |
| Couch-to-5K | The Active Network, Inc. | 11 | 19 |  | 2.59 |
| Two Hundred Situps | SoftwareX | 11 | 26 | 4 | 2.59 |
| Calorie Counter & Diet Tracker by MyFitnessPal | MyFitnessPal, LLC | 10 | 1 | 4.5 | 0 |
| Get Running (Couch to 5K) | Benjohn Barnes | 10 | 29 | 5 | 4.19 |
| MapMyRUN GPS Running | MapMyFITNESS Inc | 9 | 3 | 4 | 0 |
| MapMyRUN+ GPS Running | MapMyFITNESS Inc | 9 | 2 | 4.5 | 4.19 |
| MapMyRIDE+ GPS Cycling | MapMyFITNESS Inc | 9 | 15 | 4 | 4.19 |
| Weight Watchers Mobile AU | Weight Watchers International, Inc. | 8 | 11 | 3 | 0 |
| Fitness Buddy: 1700+ Exercise Workout Journal | Azumio Inc. | 7 | 3 | 4.5 | 1.29 |
| Nike+ Running | Nike, Inc | 6 | 2 | 4 | 0 |
| Ab Workouts Free | Feel Free Apps Pty Ltd | 5 | 5 | 3.5 | 0 |
| Fitness Budy Free | Azumio Inc. | 5 | 10 | 4 | 0 |
| Push Ups Free | Feel Free Apps | 5 | 28 | 3 | 0 |
| Ab Workouts Pro | Feel Free Apps | 5 | 24 | 4.5 | 1.29 |
| Leg Workouts Free | Feel Free Apps | 4 | 9 | 4 | 0 |
| Arm Workouts Free | Feel Free Apps | 4 | 15 | 4 | 0 |
| Core Workouts Free | Feel Free Apps | 4 | 17 |  | 0 |
| But Workouts Free | Feel Free Apps | 4 | 19 |  | 0 |
| Hip & Thigh Workouts Free | Feel Free Apps | 4 | 23 |  | 0 |
| Daily Ab Workout FREE | Daniel Miller | 4 | 24 | 4 | 0 |
| Chest Workouts Free | Feel Free Apps | 4 | 29 |  | 0 |
| Arm Workouts Pro | Feel Free Apps | 4 | 18 |  | 1.29 |
| Cardio Workouts Free | Feel Free Apps | 3 | 6 |  | 0 |
| Calorie Counter New Zealand - Easy Diet Diary | Xyris Holdings Pty Ltd | 3 | 8 |  | 0 |
| Fitspiration | Swift Fox Labs P/L | 3 | 17 | 1 | 1.29 |
| Shoulder Workouts Pro | Feel Free Apps | 3 | 21 |  | 2.59 |
| Weightbot - Track your Weight in Style | Tapbots | 2 | 28 | 4.5 | 2.59 |
| *Note*. BCTs = Behaviour Change Techniques. | | | | | |
